# MANF protects human pancreatic beta cells against stress-induced cell death

**DOI:** 10.1007/s00125-018-4687-y

**Published:** 2018-07-21

**Authors:** Elina Hakonen, Vikash Chandra, Christopher L. Fogarty, Nancy Yiu-Lin Yu, Jarkko Ustinov, Shintaro Katayama, Emilia Galli, Tatiana Danilova, Päivi Lindholm, Aki Vartiainen, Elisabet Einarsdottir, Kaarel Krjutškov, Juha Kere, Mart Saarma, Maria Lindahl, Timo Otonkoski

**Affiliations:** 1 Research Programs Unit, Molecular Neurology, Biomedicum Helsinki, University of Helsinki, PO Box 63, (Haartmaninkatu 8), 00014 Helsinki, Finland; 2 Children’s Hospital, University of Helsinki and Helsinki University Central Hospital, Helsinki, Finland; 3 Clinicum, Faculty of Medicine, University of Helsinki, Helsinki, Finland; 4 Department of Biosciences and Nutrition, Karolinska Institutet, Huddinge, Sweden; 5 Research Program in Developmental Biology, Institute of Biotechnology, University of Helsinki, Helsinki, Finland; 6 The Folkhälsan Institute of Genetics, Helsinki, Finland; 7 Competence Centre on Health Technologies, Tartu, Estonia; 8 Department of Medical and Molecular Genetics, King’s College London, London, UK

**Keywords:** Beta cell proliferation, Beta cell protection, Endoplasmic reticulum stress, Mesencephalic astrocyte-derived neurotrophic factor (MANF), NF-κB

## Abstract

**Aims/hypothesis:**

There is a great need to identify factors that could protect pancreatic beta cells against apoptosis or stimulate their replication and thus prevent or reverse the development of diabetes. One potential candidate is mesencephalic astrocyte-derived neurotrophic factor (MANF), an endoplasmic reticulum (ER) stress inducible protein. *Manf* knockout mice used as a model of diabetes develop the condition because of increased apoptosis and reduced proliferation of beta cells, apparently related to ER stress. Given this novel association between MANF and beta cell death, we studied the potential of MANF to protect human beta cells against experimentally induced ER stress.

**Methods:**

Primary human islets were challenged with proinflammatory cytokines, with or without MANF. Cell viability was analysed and global transcriptomic analysis performed. Results were further validated using the human beta cell line EndoC-βH1.

**Results:**

There was increased expression and secretion of MANF in human beta cells in response to cytokines. Addition of recombinant human MANF reduced cytokine-induced cell death by 38% in human islets (*p* < 0.05). MANF knockdown in EndoC-βH1 cells led to increased ER stress after cytokine challenge. Mechanistic studies showed that the protective effect of MANF was associated with repression of the NF-κB signalling pathway and amelioration of ER stress. MANF also increased the proliferation of primary human beta cells twofold when TGF-β signalling was inhibited (*p* < 0.01).

**Conclusions/interpretation:**

Our studies show that exogenous MANF protein can provide protection to human beta cells against death induced by inflammatory stress. The antiapoptotic and mitogenic properties of MANF make it a potential therapeutic agent for beta cell protection.

**Electronic supplementary material:**

The online version of this article (10.1007/s00125-018-4687-y) contains peer-reviewed but unedited supplementary material, which is available to authorised users.

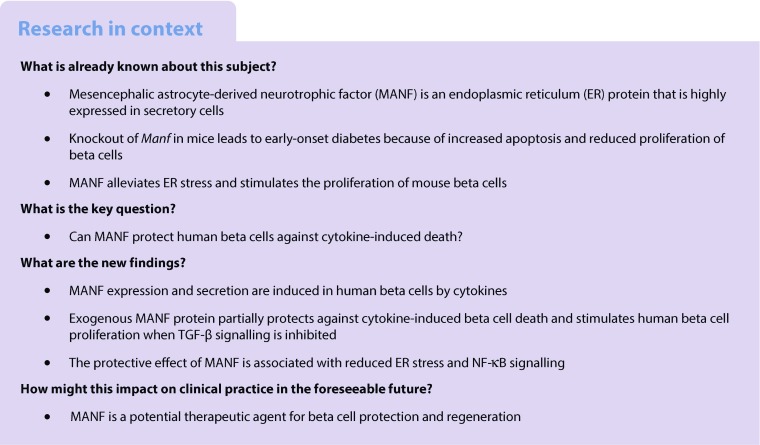



## Introduction

Insulin-secreting pancreatic beta cells are long-lived and thus vulnerable to various metabolic and cytotoxic assaults. In the case of type 1 diabetes, beta cells are selectively targeted by autoimmune attack and die mostly through apoptosis. Proinflammatory cytokines such as IL-1β, IFN-γ, IL-17 and TNF-α have been shown to be crucial players in this process [1, 2]. There is a great need to identify factors that could protect beta cells against apoptosis, stimulate beta cell proliferation and thus prevent or reverse the development of diabetes.

Mesencephalic astrocyte-derived neurotrophic factor (MANF) is a small protein with a molecular mass of 18 kDa. It contains an amino-terminal signal peptide that directs it to the endoplasmic reticulum (ER) and, when cleaved, results in a mature protein that can be secreted [3]. Interestingly, at the carboxy-terminal end MANF has an RTDL motif that is responsible for its retention in the ER and fine-tunes MANF secretion [3]. MANF was originally discovered as a survival-promoting factor for brain dopaminergic neurones in vitro [4] and in vivo [5]. Since then it has also been shown to protect several other cell types, including neuronal cells, cardiomyocytes and HeLa cells [3, 6–8]. Despite extensive studies, the receptors for MANF remain unknown [3]. It has been suggested that MANF binds via its C-terminal KDEL-like motif RTDL to the KDEL receptor on the cell surface [9]. It has recently been reported that MANF binds to lipid sulfatides (e.g. 3-*O*-sulfogalactosylceramide) and promotes MANF uptake and cytoprotection [10]. The protective effect of MANF probably depends on its ability to alleviate ER stress [5, 7]. We previously reported that *Manf* knockout mice used as a model of diabetes develop the condition owing to a progressive postnatal reduction of beta cell mass caused by reduced beta cell proliferation and increased beta cell apoptosis [11]. Additionally, both in vitro and in vivo, MANF was identified as a mitogen for mouse beta cells. Furthermore, a recent study by Cunha et al [12] showed that thrombospondin 1 protects rat, mouse and human beta cells against cytokine-induced cell death by maintaining the expression of MANF.

Unresolved ER stress and chronic activation of the unfolded protein response (UPR), a cell signalling pathway involved in the restoration of ER homeostasis, are involved in beta cell dysfunction and death in the pathogenesis of both type 1 and type 2 diabetes [13, 14]. We demonstrated increased expression of UPR markers and sustained phosphorylation of the eukaryotic initiation factor 2 alpha (eIF2α), which leads to global protein synthesis arrest, in islets from *Manf* knockout mice [11]. The mechanism by which lack of MANF induces sustained ER stress in beta cells remains elusive, as does the potential protective effect of this growth factor, particularly when administered as an extracellular protein.

In this study, we tested whether human MANF protein could protect primary and clonal human beta cells against death induced by proinflammatory cytokines. Global transcriptomic analysis was performed to identify molecular mechanisms behind the observed partially protective effects of MANF.

## Methods

### Human islets

Two formalin-fixed, paraffin-embedded pancreatic samples were received from the PanFin network [15] and one from an autopsy at the Helsinki University Central Hospital. Islets were isolated from cadaveric organ donors at the central laboratory of the Nordic Network for Islet Transplantation in Uppsala, Sweden, and distributed through the European Consortium for Islet Transplantation (ECIT). The use of human islets was approved by the Ethics Committee of the Children’s Hospital, University of Helsinki. Details of the formalin-fixed, paraffin-embedded pancreatic samples and organ donor characteristics are presented in electronic supplementary material (ESM) Table 1. In the cytokine experiments, islets from some donors were used for RNA sequencing (RNA-seq) while those from other donors were used for additional validation of the results (cell death and quantitative reverse transcription PCR [qRT-PCR]). Samples selected for RNA-seq were chosen based on the purity of the original preparation (>50% by dithizone staining) and induction of apoptosis by cytokines (a more than twofold increase in cell death). The islets were shipped to Helsinki within 2–8 days after isolation. Upon arrival the islets were cultured in Ham’s-F10 medium supplemented with 0.5% (vol./vol.) BSA, penicillin (100 U/ml) and streptomycin (172 μmol/l) on non-adherent culture plates.

### EndoC-β cells

We cultured the EndoC-βH1 cells as previously described [16]. EndoC-βH3 cells were obtained from Univercell-Biosolution (Toulouse, France), were negative for mycoplasma and were cultured as per their instructions. These cells were used for proliferation studies after excision of the immortalising transgenes by tamoxifen treatment [17]. See the ESM for further details.

### Immunostaining and immunoblotting of EndoC-βH1 cells

For immunofluorescence, cells were cultured on fibronectin–Matrigel-coated (both from Sigma-Aldrich, St. Louis, MO, USA) plates. After treatment, cells were fixed with 4% (wt/vol.) paraformaldehyde (PFA) and permeabilised in 0.5% (vol./vol.) Triton-X-100 (Sigma-Aldrich) before being incubated overnight with primary antibodies (ESM Table 2). For immunoblotting, cells were lysed in Laemmli buffer, and proteins were resolved by Any kD Mini-PROTEAN TGX gel (Bio-Rad, Hercules, CA, USA). See the ESM for further details.

### Cytokines

The cytokine concentrations used were based on our previous experiments on human islets [18]. Islets and EndoC-βH1 cells were stimulated either with a cytokine cocktail consisting of IL-1β (5 ng/ml) and IFN-γ (50 ng/ml) (cytokine cocktail I) or with a more potent cytokine cocktail consisting of IL-1β (5 ng/ml), IFN-γ (50 ng/ml), IL-17 (100 ng/ml) and TNF-α (10 ng/ml), all from R&D Systems (Minneapolis, MN, USA) (cytokine cocktail II), with or without MANF (100 ng/ml). Recombinant human MANF protein was produced in CHO cells (no. P-101-100; Icosagen, Tartumaa, Estonia). The cytokine stimulations were performed in Ham’s-F10 medium supplemented with 0.5% (vol./vol.) BSA using the cytokine concentrations described above. RNA samples were collected after 48 h and cell death was analysed after 72 h of exposure to cytokines.

### qRT-PCR

cDNA was synthesised using the random hexamer primer of the High Capacity cDNA Reverse Transcription kit according to the manufacturer’s recommendations (Applied Biosystems, Foster City, CA, USA). SYBR Green JumpStart Taq Ready Mix for qRT-PCR (Sigma-Aldrich) was used for the reactions with a Corbett Rotor-Gene 6000 (Qiagen, Hilden, Germany). The median C_t_ values were used for ${2}^{-\Delta \Delta {\mathrm{C}}_{\mathrm{t}}}$ analysis. Cyclophilin G (*PPIG*) was used as an endogenous control. An exogenous positive control was used as a calibrator. See the ESM for further details. Primer sequences for *MANF*, *GRP78* (also known as *HSPA5*), *CHOP* (also known as *DDIT3*), *sXBP1*, *ATF4*, *ATF6*, *ATF3*, *PreINS*, *INS*, *PDX1*, *MAFA*, Cyclophilin G, *BCL10*, *Ki67*, *CDK1* and *CDK4* are presented in ESM Table 3.

### MANF assay

For quantification of secreted MANF protein, the conditioned medium samples were analysed on an in-house sandwich ELISA specific for human MANF as previously described [19]. See the ESM for further details.

### Assessment of cell viability after exposure to cytokines

For cell viability analysis, partially trypsinised islets were allowed to attach for 48 h in RPMI 1640 supplemented with 10% (vol./vol.) FCS before starting the experiment. After 72 h of stimulation with cytokines, the cells were stained with 5 μg/ml propidium iodide (PI; Sigma-Aldrich) for 30 min at 37°C. The cells were then fixed in 4% (wt/vol.) PFA for 20 min and subsequently immunostained for C-peptide. The number of dead beta cells was determined by calculating the PI-positive area within the C-peptide-positive area. In our experimental set-up, cells may be superimposed, and thus these observations should be considered as semi-quantitative. Analyses were performed using Image-Pro Analyzer 6.0 (Media Cybernetics, Bethesda, MD, USA) and the analyser was blind to the sample identity.

### Quantification of annexin-V/PI staining in EndoC-βH1 cells

Apoptotic cells were quantified with BD Annexin-V-FITC Apoptosis Detection Kit (BD-Biosciences, Franklin Lakes, NJ, USA) following the manufacturer’s instructions. See the ESM for further details.

### siRNA transfection in EndoC-βH1 cells

EndoC-βH1 cells were transfected using lipofectamine RNAiMAX (Life Technologies, Thermo Fisher Scientific, Waltham, MA, USA) and ON-TARGET*plus* small interfering (si)RNA SMARTpool for human *MANF* gene (40 nmol/l; si*MANF*) or ON-TARGET*plus* Non-targeting-pool (non-target siRNA [siNT]) (Dharmacon, Lafayette, CO, USA) as previously described [20]. Cells were analysed 72 h (48 h + 24 h cytokine treatment) after transfection for apoptosis or ER stress-associated markers.

### RNA transcriptome and data analysis

RNA-seq data were analysed using the STRTprep version v3dev pipeline (https://github.com/shka/STRTprep; commit 6389622) as described elsewhere [21]. Differential expression analysis was performed using SAMstrt [22]. Gene Ontology (GO) over-representation analysis was performed using GOrilla [23]. See the ESM for further details.

### Beta cell proliferation assay

For 5-ethynyl-2′-deoxyuridine (EdU) labelling the islets were adhered to culture plates as described for the cell viability analysis. The proliferation experiments were performed in RPMI-1640 supplemented with 10% (vol./vol.) FCS. MANF (100 ng/ml), TGF-β inhibitor SB431542 (2 μmol/l; Selleck Chemicals, Munich, Germany) and EdU (100 μmol/l; Invitrogen, Thermo-Fisher Scientific, Waltham, MA, USA) were added at time zero; 50% of the media was changed every 48 h. After 96 h, the cells were fixed in 4% (wt/vol.) PFA for 20 min and EdU detected by Click-iT EdU Imaging Kit (Invitrogen) according to the manufacturer’s protocol, followed by C-peptide staining. The number of proliferating C-peptide-positive cells was counted from at least five randomly chosen images per donor or by counting all existing beta cells on the culture dish. Each image contained approximately 100–400 beta cells.

### Statistical methods

No systematic randomisation methods were applied. Blinding was applied for all quantitative immunohistochemical analyses. The results are presented as the mean ± SD from at least three independent experiments. Student’s unpaired *t* test was used to compare differences between two groups. Differences between more than two groups were calculated using the one-way ANOVA or one-way repeated measures ANOVA followed by Tukey’s test, using GraphPad Prism 6 software (La Jolla, CA, USA).

## Results

### MANF is expressed in human beta cells, and expression is upregulated in response to cytokine-induced cellular stress

**Fig. 1:**
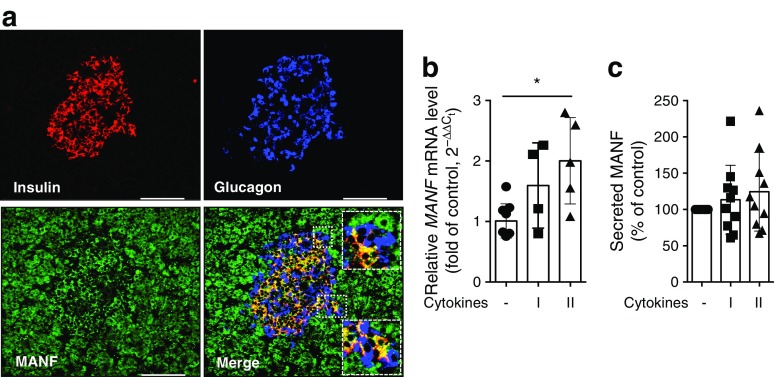
Expression of MANF in human pancreatic islets. (**a**) Immunohistochemical analysis of formalin-fixed human adult organ donor pancreatic section stained for MANF (green) and counterstained with insulin (red) and glucagon (blue). Scale bars, 100 μm. MANF immunoreactivity can be detected throughout the exocrine tissue. High-magnification inserts demonstrate complete co-localisation of MANF and insulin immunoreactivity, but a lack of co-localisation with glucagon immunoreactivity. (**b**) *MANF* mRNA expression by qRT-PCR from human islets treated with cytokines for 48 h (*n* = 4–5). (**c**) Relative MANF protein secretion from human islets treated with cytokines quantified by ELISA after 72 h (*n* = 10). Equal numbers of islets were plated in each condition. qRT-PCR data were normalised to the housekeeping gene cyclophilin G. Cytokine cocktail I, IL-1β + IFN-γ; cytokine cocktail II, IL-1β + IFN-γ + IL-17 + TNF-α. Data represent the mean ± SD of at least three independent experiments. *p < 0.05 (one-way ANOVA followed by Tukey’s test)

**Fig. 2:**
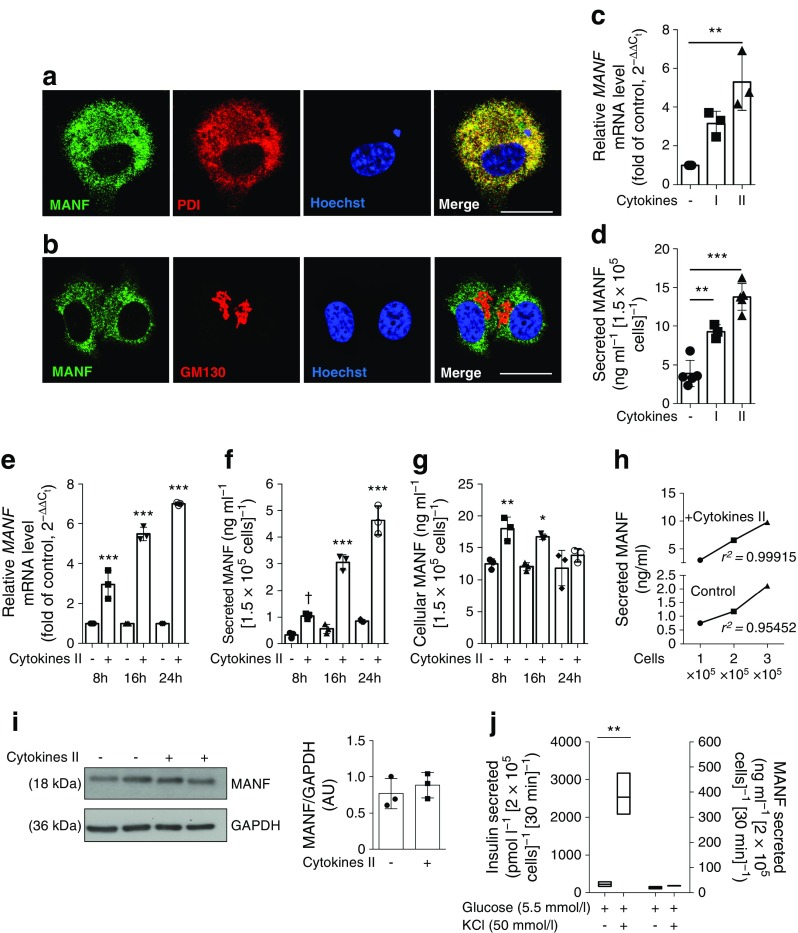
Cytokines induce MANF expression and secretion in EndoC-βH1 cells. (**a**, **b**) Immunofluorescence analysis for co-localisation of MANF (green) and (**a**) PDI (an ER marker; red) and (**b**) GM130 (a Golgi marker, red) in EndoC-βH1 cells. Nuclei were stained with Hoechst (blue). Scale bars, 25 μm. (**c**) *MANF* mRNA expression by qRT-PCR and (**d**) MANF secretion in supernatant fractions from culture, quantified by ELISA in EndoC-βH1 cells stimulated with cytokine cocktail I or II for 24 h and 48 h, respectively. (**e**–**g**) EndoC-βH1 (1.5 × 10^5^ seeded) cells were treated with cytokine cocktail II for a time course of 8 h, 16 h and 24 h and analysed for (**e**) *MANF* mRNA expression, (**f**) secreted MANF in the supernatant fraction and (**g**) cellular MANF normalised to total protein content. Statistical comparisons are between the cytokine treated vs control condition of the same timepoint. (**h**) 1 × 10^5^, 2 × 10^5^ and 3 × 10^5^ cells were treated with cytokine cocktail II for 24 h. Secreted MANF in the supernatant fraction was quantified with ELISA. (**i**) Western blot analysis of MANF protein and densitometric analysis for band intensities normalised to glyceraldehyde 3-phosphate dehydrogenase (GAPDH) in EndoC-βH1 cells treated with cytokine cocktail II for 24 h. (**j**) EndoC-βH1 cells were stimulated with either 5.5 mmol/l glucose alone or in presence of 50 mmol/l KCl for 30 min, and the supernatant fraction was quantified for both insulin and MANF protein using ELISA. qRT-PCR data were normalised to the housekeeping gene cyclophilin G. Cytokine cocktail I, IL-1β + IFN-γ; cytokine cocktail II, IL-1β + IFN-γ + IL-17 + TNF-α. Data represent the mean ± SD of at least three independent experiments. *p < 0.05, **p < 0.01, ***p < 0.001; ^†^*p* = 0.0507 (one-way ANOVA followed by Tukey’s test). AU, arbitrary units

Strong MANF immunoreactivity was detected in human insulin-positive cells as well as in the exocrine pancreas (Fig. 1a and ESM Fig. 1; donor details in ESM Table 1). The specificity of the MANF antibody was confirmed by the negative or very faint staining in *Manf* knockout mouse tissues [11] or EndoC-βH1 cells after siRNA knockdown of MANF expression. Interestingly, glucagon cells and pancreatic polypeptide cells were negative for MANF (Fig. 1a and ESM Fig. 1c, d). MANF expression was also detected in human fetal pancreatic epithelium at day 72 (ESM Fig. 1a, b). At this stage, the insulin and glucagon double-positive cells were also expressing MANF. Furthermore, high MANF expression was detected in EndoC-βH1 cells (Fig. 2a, b). In these cells, MANF was mostly localised to the ER, as shown by MANF and protein disulfide isomerase (PDI) double immunohistochemistry (Fig. 2a).

*MANF* mRNA expression in human islets increased in response to IL-1β, IFN-γ, IL-17 and TNF-α, although the response to IL-1β and IFN-γ alone did not reach statistical significance (Fig. 1b). A potent increase in *MANF* mRNA expression was also detected in EndoC-βH1 cells in response to the more potent cytokine cocktail II, whereas the response to the minor cocktail I did not reach statistical significance (Fig. 2c). MANF release was analysed by ELISA from human islet and EndoC-βH1 cell culture supernatant fractions. In the primary islets, there was a trend of increased secretion upon cytokine exposure that did not reach statistical significance (Fig. 1c). However, MANF secretion from EndoC-βH1 cells doubled when the cells were stressed with IL-1β and IFN-γ, and tripled when they were stimulated with the more potent cytokine cocktail II (Fig. 2d). Furthermore, a time course experiment in EndoC-βH1 cells showed a progressive cytokine-induced increase of MANF expression and secretion, while the total cellular MANF content did not increase similarly (Fig. 2e–g). This indicates that most of the newly synthesised MANF was secreted into the medium. A linear correlation was found between MANF secretion and cell number (Fig. 2h). In accordance with the measurement of total cellular content, a slight non-significant increase of MANF protein was observed by Western blot at 24 h of cytokine stimulation (Fig. 2i). Stimulation of EndoC-βH1 cells with 50 mmol/l KCl resulted in an 11-fold (*p* = 0.002) increase in insulin secretion with no concurrent change in MANF secretion (Fig. 2j), demonstrating that MANF is not released together with insulin.

### MANF partially prevents cytokine-induced beta cell death

**Fig. 3:**
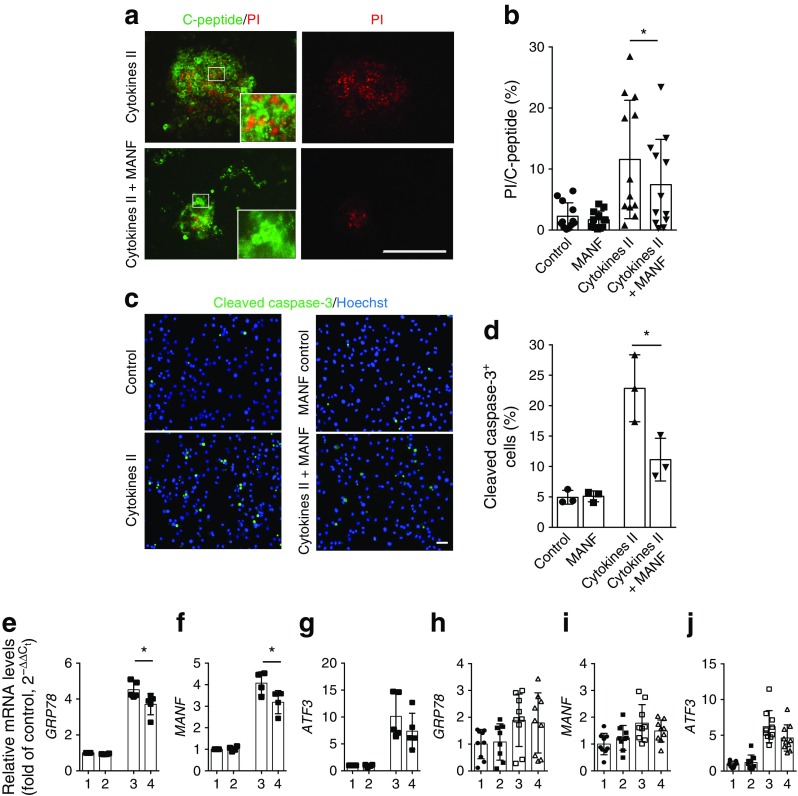
MANF partially prevents human beta cell death induced by cytokines and ameliorates cytokine-induced ER stress. (**a**, **b**) Human islets from 12 donors were cultured for 72 h with cytokine cocktail II (IL-1β + IFN-γ + IL-17 + TNF-α) in the presence or absence of MANF, and cell death was analysed by percentage of the PI-positive area within the C-peptide-positive area. (**a**) Representative images of cytokine-treated islets as indicated, showing the fluorescent signal for C-peptide (green) and PI (red), with higher magnification inserts. Scale bar, 200 μm. (**b**) Quantification of the PI-positive area within the C-peptide-positive area. *p < 0.05 (one-way repeated measures ANOVA followed by Tukey’s test). (**c**) Apoptosis of EndoC-βH1 cells revealed by immunostaining of cleaved caspase-3 after 24 h of treatment with MANF alone or cytokine cocktail II with MANF. Nuclei were stained with Hoechst (blue). Scale bar, 50 μm. (**d**) Quantification of apoptotic cells in the same experiment (*n* = 3). (**e**–**g**) qRT-PCR analysis for expression of the UPR-related genes *GRP78*, *MANF* and *ATF3* in EndoC-βH1 cells and (**h**–**j**) in human pancreatic islets (*n* = 9–10) in a similar experiment. qRT-PCR data were normalised to the housekeeping gene cyclophilin G. On the *x*-axis: 1, control; 2, MANF; 3, cytokine cocktail II; 4, cytokine cocktail II + MANF. Data are represented as the mean ± SD of at least three independent experiments. *p < 0.05 (one-way ANOVA followed by Tukey’s test)

Islets from 12 organ donors were exposed to cytokine cocktail II with or without MANF. The characteristics of the donors and islet preparations are presented in ESM Table 1. As expected, cytokines markedly increased beta cell death in isolated human islets at 72 h; these showed 11.6% cell death, a proportion that was 5.2-fold over that of control cells. MANF reduced the cytokine-induced cell death rate to 7.1% dead cells (38% inhibition, *p* < 0.05; Fig. 3a, b). Similar results were obtained with EndoC-βH1 cells. After treatment with cytokine cocktail II for 24 h, the percentage of apoptotic cells was 23 ± 3.2%, and this was reduced by 50% (down to 11 ± 2.0%; *p* < 0.05) in the presence of MANF protein (Fig. 3c, d).

MANF significantly reduced the upregulation of *GRP78* (also known as *HSPA5* and *BIP*) and *MANF* mRNA upon cytokine stimulation in EndoC-βH1 cells (Fig. 3e–f). Other UPR markers, including *ATF3*, *ATF4* and *CHOP* (also known as *DDIT3*), did not decrease significantly (Fig. 3g, and data not shown). Expression of the beta cell-specific genes *INS*, *PDX1* and *MAFA* was not affected by the addition of recombinant MANF (ESM Fig. 2a). Insulin secretion and insulin content were unaffected in EndoC-βH1 cells after the addition of recombinant MANF (ESM Fig. 2b–d). Overall, the upregulation of ER stress genes was more modest in human islets than in EndoC-βH1 cells (Fig. 3h–j).

### Knockdown of MANF in EndoC-βH1 cells results in aggravated ER stress and activation of UPR pathways

**Fig. 4:**
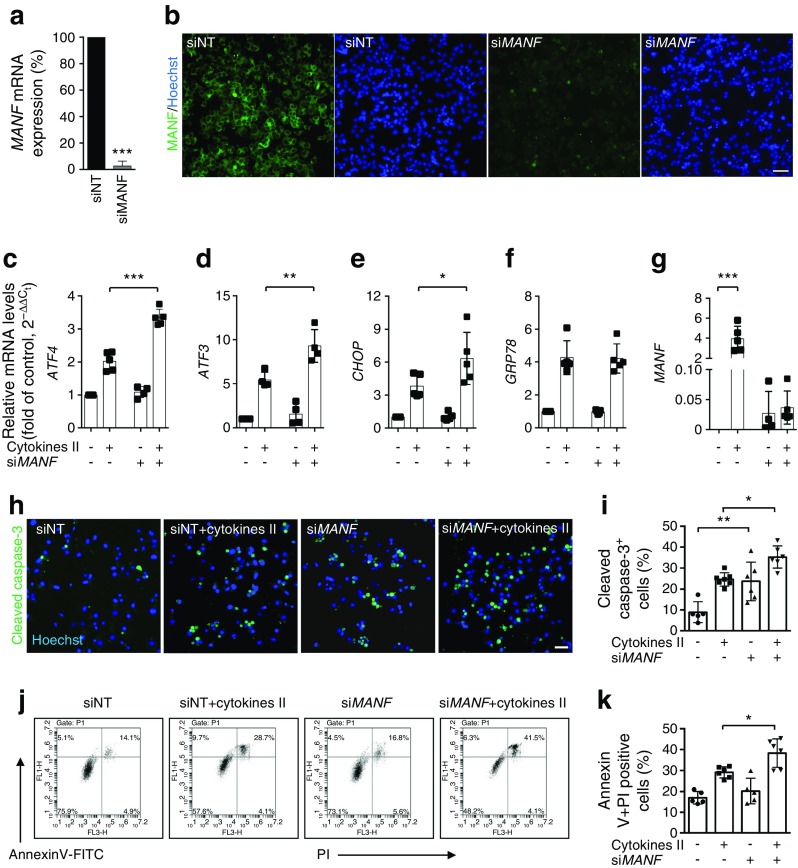
Knockdown of MANF in EndoC-βH1 cells exacerbates cytokine-induced ER stress and apoptosis. EndoC-βH1 cells were transfected with control siNT or si*MANF* and analysed at 72 h after transfection. (**a**) *MANF* mRNA expression determined by qRT-PCR, presented relative to MANF expression in siNT-transfected cells. (**b**) Immunofluorescence detection of MANF (green) in siNT- and si*MANF*-transfected cells. Nuclei were stained with Hoechst (blue). (**c**–**g**) qRT-PCR analysis of the UPR-related genes *ATF4*, *ATF3*, *CHOP*, *GRP78* and *MANF* in siNT- and si*MANF*-transfected cells exposed to cytokine cocktail II or left untreated for 24 h. qRT-PCR data were normalised to cyclophilin G and plotted as the fold of siNT control cells. (**h**) Representative immunofluorescence images of cleaved caspase-3 in siNT- and si*MANF*-transfected cells left untreated or treated with cytokine cocktail II for 24 h, at 48 h after siRNA transfection. (**i**) Quantification of apoptotic cells. (**j**) Flow cytometry analysis of apoptotic cells based on annexin-V and PI in siNT- and si*MANF*-transfected cells treated for 24 h with or without cytokine cocktail II. (**k**) Quantification of annexin-V and PI double-positive cells. Scale bars, 50 μm. Data are presented as the mean ± SD of at least three independent experiments. *p < 0.05, **p < 0.01, ***p < 0.001 (one-way ANOVA, followed by Tukey’s test)

To investigate the importance of MANF in human beta cells, we used siRNA-based loss of function in EndoC-βH1 cells. We achieved efficient knockdown of *MANF* mRNA (>90%) (Fig. 4a) and protein (Fig. 4b). This led to a significantly increased expression of UPR-associated genes such as *ATF4*, *ATF3* and *CHOP* when the cells were exposed to cytokines (Fig. 4c–g). The aggravated stress responses were also reflected in increased apoptosis as revealed by cleaved caspase-3 immunostaining (Fig. 4h, i) and by FACS analysis of annexin-V- and PI-stained cells (Fig. 4j, k).

### RNA transcriptome analysis

To assess the mechanism of the MANF-induced protective effect on human beta cells, full transcriptome analysis was performed in samples from six islet preparations stimulated with cytokine cocktail II with or without MANF. For RNA-seq, we used the single-cell tagged reverse transcription method with external spike-in controls for normalisation [21, 22].

**Fig. 5:**
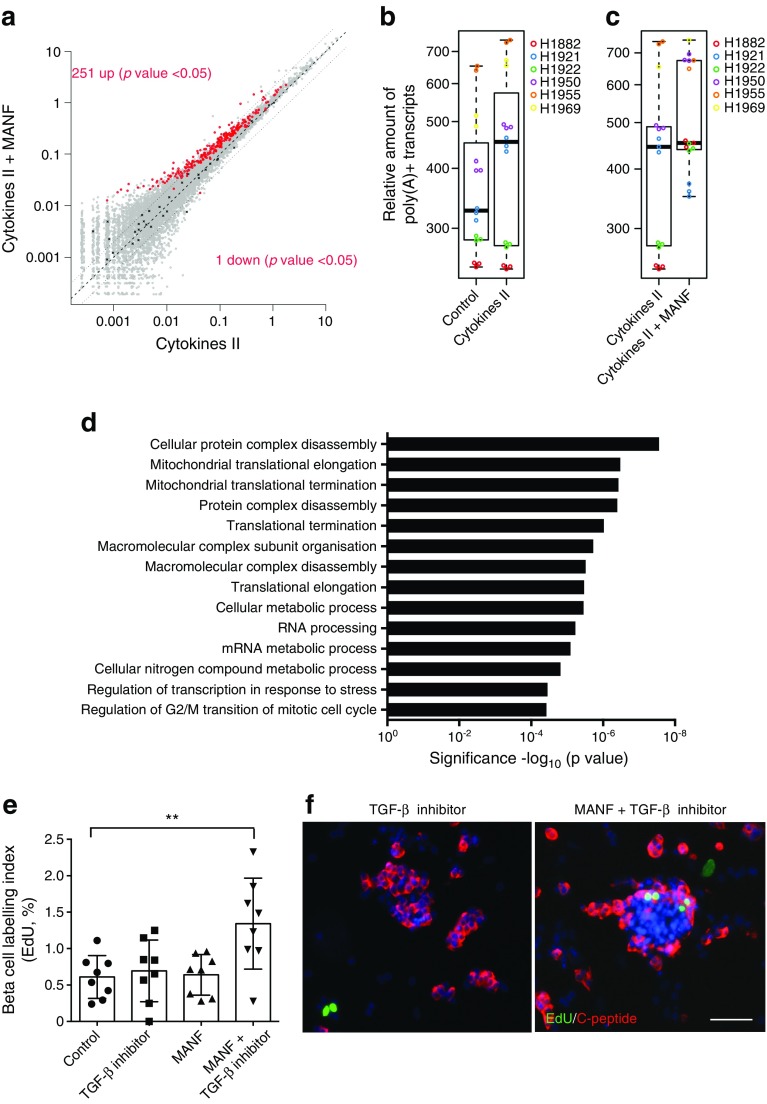
MANF induces a global upregulation of gene expression and induces proliferation of human beta cells with simultaneous TGF-β inhibition. (**a**) Scatter plot of relative gene expression results based on spike-in normalisation for cytokine cocktail II vs cytokine cocktail II + MANF treatment of human islet cells. The *x*- and *y*-axes depict relative expression levels, plotted on a log scale. The central dashed line marks equal gene expression. The two dotted lines mark the ± twofold boundaries. Grey dots represent all the genes detected in the expression profiling experiment. Red dots mark the significantly differentially expressed genes, and black crosses mark the spike-in molecules. (**b**, **c**) Tukey box plots showing the relative amount of poly(A)+ transcripts. The thick centre line represents the statistical median. The lower and upper lines of the boxes represent the 1st and 3rd quartiles. The upper and lower whiskers denote the furthest points that are not outliers. (**b**) Comparison between control and cytokine-treated islets. (**c**) Comparison between cytokine and cytokine + MANF-treated samples. Individual donors are represented by different colours. (**d**) GO enrichment analysis of upregulated genes (FDR <0.05) for cytokines + MANF compared with cytokines alone. (**e**, **f**) Human islets from eight organ donors were cultured for 96 h with MANF (100 ng/ml), TGF-β inhibitor SB431542 (2 μmol/l) or both. EdU was added to the culture medium at the start of the experiment. (**e**) Quantification of proliferating beta cells analysed by EdU and C-peptide double-positive cells relative to the total number of C-peptide-positive cells. **p < 0.01 (one-way ANOVA followed by Tukey’s test). (**f**) Representative C-peptide (red) and EdU (green) double immunofluorescence in islets stimulated by SB431542 or SB431542 + MANF. Scale bar, 50 μm

Addition of MANF alone did not lead to any significant changes in gene expression (data not shown). With the cytokines alone, 618 genes were significantly upregulated while 377 genes were downregulated compared with the control. Overall, the cytokine-induced changes were similar to those previously reported [24]. Surprisingly, when the islets were stimulated with cytokines together with MANF, a global increase in gene expression was detected when compared with cytokines alone. The number of total mRNA reads increased by 15% (Fig. 5a–c). In total, 251 genes were significantly upregulated and only one gene was downregulated upon addition of MANF (Fig. 5a). Interestingly, the only significantly downregulated gene was *BCL10*, a known inducer of apoptosis and an upstream regulator of NF-κB signalling [25, 26].

### MANF induces proliferation of human beta cells

GO analysis of the upregulated genes revealed significant over-representation for genes with functions related to gene expression, protein complex disassembly, cellular metabolic processes and regulation of G2/M transition during the mitotic cell cycle (Fig. 5d). To test whether MANF could stimulate the replication of human beta cells, we stimulated human primary islets with MANF and analysed beta cell proliferation via EdU incorporation. However, no changes could be detected with MANF alone (Fig. 5e). It has been shown that the proliferative capacity of beta cells declines with age due to accumulation of the cell cycle inhibitor p16^Ink4a^ [27, 28]. It has recently been shown that TGF-β inhibitors can repress the Ink4a/Arf locus, resulting in increased beta cell proliferation in transplanted human islets [29]. Pharmacological inhibition of TGF-β signalling with SB431542 did not affect beta cell proliferation. However, simultaneous addition of MANF and SB431542 induced a significant twofold increase in beta cell proliferation in vitro (Fig. 5e, f). To further confirm the mitogenic effect of MANF on beta cells, we used the third-generation human EndoC beta cell line EndoC-βH3 [17]. Treatment of the cells with tamoxifen for 3 weeks resulted in a loss of SV40 large T antigen on immunocytochemistry and proliferative quiescence (ESM Fig. 3a). Treatment of the SV40 large T antigen-excised EndoC-βH3 cells with MANF alone resulted in a threefold increase in proliferation, which was not potentiated by SB431542 (ESM Fig. 3b–d).

### MANF affects NF-κB pathway signalling in human beta cells

**Fig. 6:**
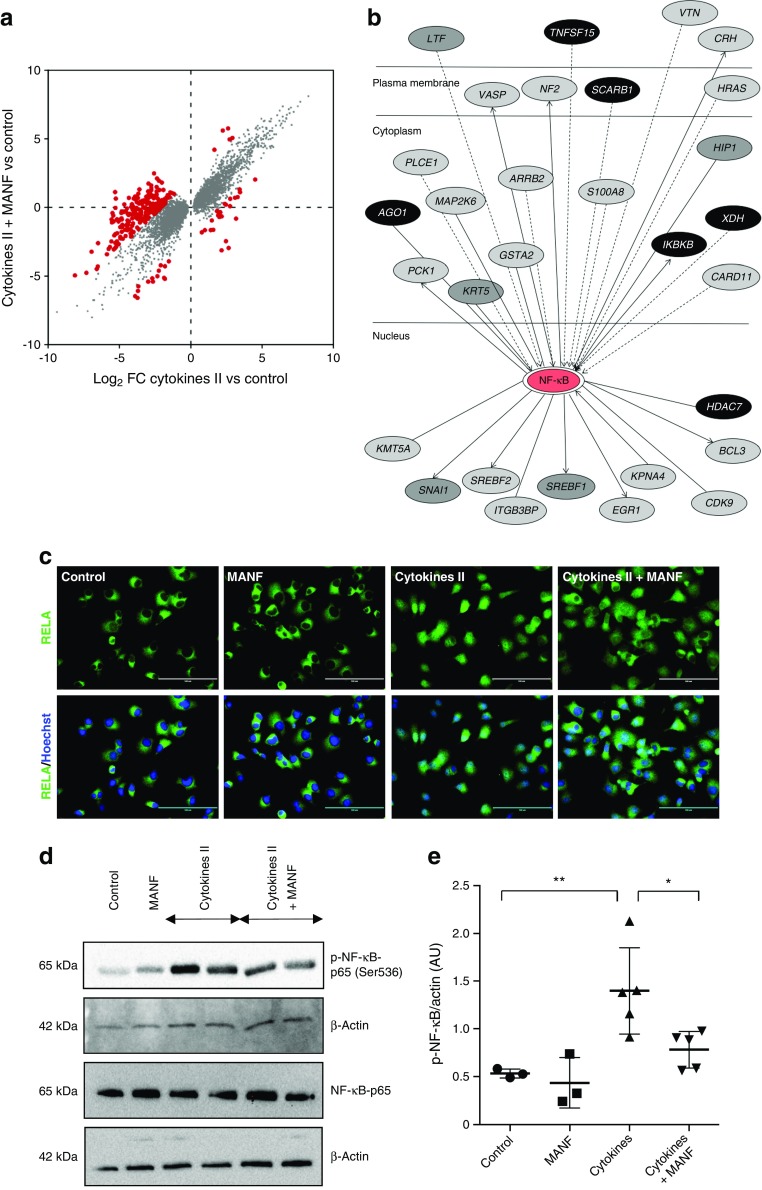
Recombinant MANF treatment inhibits NF-κB activity in beta cells. (**a**) Significantly differentially expressed transcripts (FDR <0.05) in cytokines + MANF vs control, plotted against cytokines vs control. Grey dots mark the genes that responded similarly in the two conditions. Red dots mark the 282 genes that responded differentially at least by log_2_ FC2. (**b**) The 282 genes that responded differently in (**a**) were analysed using ingenuity pathway analysis. Thirty genes were identified as being related to the NF-κB pathway. Interaction network of NF-κB-regulated genes differentially expressed by MANF. Black ovals, downregulated genes; grey ovals, upregulated genes, with lighter shades of grey signifying more strongly upregulated genes. (**c**) Immunofluorescence analysis of the RELA (NF-κB p65) component of the NF-κB complex in EndoC-βH1 cells following treatment with MANF, cytokine cocktail II, or cytokine cocktail II + MANF for 8 h. Note the nuclear accumulation of RELA induced by cytokines and its inhibition by MANF. Nuclei were stained with Hoechst (blue). Scale bars, 25 μm. (**d**) Western blot analysis of phospho-NF-κB p65 (Ser536) and NF-κB p65, with the respective loading control β-actin. (**e**) Densitometric quantification of the Western blot bands of phospho-NF-κB p65 (Ser536) vs β-actin. Data are presented as the mean ± SD of at least three independent experiments. *p < 0.05, **p < 0.01 (one-way ANOVA, followed by Tukey’s test)

To identify potential genes and signalling pathways altered by MANF, we performed differential expression analysis from the RNA transcriptome data using TMM normalisation in edgeR, a standard RNA-seq analytical method which assumes that most genes are not differentially expressed between the samples [30]. After TMM normalisation, we compared genes differentially expressed in cytokines, vs cytokines with MANF treatment. We therefore plotted four cytokines vs control against four cytokines and MANF vs control and found 282 differentially expressed genes (log_2_ fold change [FC]2, false discovery rate [FDR] <0.05) (Fig. 6a, ESM Table 4). These 282 genes were analysed using Ingenuity pathway analysis (Ingenuity Systems, Redwood City, CA, USA) showing enrichment for genes related to cellular movement (47 genes), cellular proliferation and growth (27 genes), gene expression (63 genes) and the cell cycle (16 genes). Interestingly, of the 282 genes affected by MANF, 30 were found to encode genes that interact with NF-κB (Fig. 6b). Because of the known central role of this pathway in the inflammatory responses, including in beta cells [31, 32], we chose to specifically investigate it further in EndoC-βH1 cells.

### MANF inhibits cytokine-induced NF-κB activation in EndoC-βH1 cells

Nuclear translocation and phosphorylation at the serine 536 site of RELA/p65 are hallmarks of NF-κB pathway activation [33, 34]. In the presence of MANF the translocation of the RELA/p65 component of the NF-κB complex to the nucleus was clearly inhibited (Fig. 6c). Consistent with this result, immunoblotting analysis showed a significant reduction (around 46%) in the expression of phospho-NF-κB p65 (Ser536) upon addition of MANF to cytokines, indicating inhibition of NF-κB activation [34] (Fig. 6 d, e). *BCL10*, a caspase recruitment domain (CARD)-containing upstream regulator of NF-κB signalling was significantly downregulated by addition of MANF in cytokine-treated islets [25]. We found that cytokines induced a twofold increase of *BCL10* expression in EndoC-βH1. However, this effect was not effectively counteracted by exogenous MANF protein (ESM Fig. 4a). However, knockdown of MANF by si*MANF* significantly increased the level of *BCL10* mRNA in cytokine-treated cells (ESM Fig. 4b), indicating that MANF has a role in controlling *BCL10* expression. Finally, we also investigated other potentially important signalling pathways, mitogen activated protein kinase (MAPK) and phosphatidylinositol 3-kinase (PI3K), which were induced by cytokines as reflected by increased phosphorylation of extracellular regulated kinase (ERK) and protein kinase B (Akt), respectively. However, MANF did not modulate these signalling events (ESM Fig. 5). These results suggest that extracellular MANF protein ameliorates cytokine-induced inflammatory responses in human beta cells at least partly by inhibiting NF-κB activity.

## Discussion

We previously showed that global knockout of *Manf* in mice results in early-onset diabetes owing to increased beta cell apoptosis and reduced proliferation via persistent activated ER stress-induced UPR pathways [11]. This led us to investigate the role of MANF as a potential regenerative factor in human beta cells. In the present study, we demonstrate that MANF expression and secretion is stimulated in cytokine-stimulated human beta cells. Furthermore, exogenous MANF protein protects primary and clonal human beta cells against cytokine-induced cell death and may even induce beta cell proliferation.

MANF is expressed at high levels in human beta cells and exocrine cells. Interestingly, we did not detect MANF protein in alpha or pancreatic polypeptide cells. This is in line with the immunohistochemical pattern of MANF expression in mouse islets (T. Danilova, T. Otonkoski and M. Lindahl, unpublished results). The alpha cells were unaffected in the *Manf* knockout mice, supporting the idea that MANF is not crucial for alpha cell function [11]. However, single-cell RNA-seq has shown similar *MANF* mRNA levels in human alpha and beta cells [35]. There are two possible explanations for this: either *MANF* mRNA is not translated in alpha cells, or the antibody used in this study does not recognise the form of MANF produced by these cells. We also detected MANF protein throughout the pancreatic epithelium during embryonic development. At this stage, MANF was present also in the insulin and glucagon double-positive cells. MANF seems to have no obvious function in pancreatic organogenesis, since there were no evident defects in embryonic pancreas development in the *Manf* knockout mice.

Generation of the human beta cell line EndoC-βH1 provides a valid model of human beta cells for in vitro studies [16]. These cells mirror the physiological characteristics of human primary beta cells and have also been shown to be sensitive to proinflammatory cytokines [36]. Recombinant human MANF reduced cytokine-induced cell death in EndoC-βH1 cells. This is in line with a recent study [12] showing similar protection in mouse beta cells and EndoC-βH1 cells from exogenously administered MANF. Furthermore, in our study, the addition of MANF reduced the expression of ER stress-associated genes and, interestingly, *MANF* expression was also reduced. Since *MANF* expression is regulated by activating transcription factor 6 (ATF6) and spliced X-box binding protein 1 (sXBP1), which bind to the ER stress element in the *MANF* promoter, it is likely that a lower level of UPR can explain the reduced *MANF* expression. The knockdown of MANF in these cells significantly aggravated ER stress responses. This is in line with data from knockout mice showing persistent ER stress in beta cells characterised by late embryonic upregulation of the UPR markers *sXbp1* and *Chop* followed by increased neonatal expression of *Atf4* and *Chop* and sustained pEIF2α activation in the protein kinase RNA-like endoplasmic reticulum kinase (PERK) pathway [11]. Recently, the timing, amplitude and kinetics of inositol-requiring enzyme 1 (IRE1)α and PERK activation has been found to be important for determining the cellular outcome of the UPR in beta cells [37]. Our results and those from other groups suggest that MANF is critical for determining the transition from physiological to apoptotic UPR in beta cells where MANF favours the alleviation of ER stress but only if unresolved beta cells switch to the apoptotic PERK/CHOP pathway and apoptosis. However, the exact role for MANF in the regulation of UPR remains to be determined.

NF-κB pathway activation is considered to be an important component of beta cell death induced by inflammatory cytokines [32]. It has recently been shown that MANF negatively regulates the NF-κB pathway by inhibiting p65-mediated transcriptional activation in fibroblast-like synoviocytes [38]. MANF is a secreted protein, but it is mostly localised to the lumen of the ER. In inflammation, it has been suggested that it translocates to the nucleus, where it could interfere with the binding of p65 to its target gene promoters, thus suppressing the expression of NF-κB target genes [38]. However, we did not observe nuclear translocation of MANF in EndoC-βH1 cells under cytokine treatment (data not shown). Analysis of the RNA-seq data identified repression of the NF-κB signalling pathway after addition of MANF, and specifically the downregulation of *BCL10*, an inducer of apoptosis acting upstream of NF-κB. In line with this, MANF clearly reduced the nuclear localisation and phosphorylation of RELA/p65 component of the NF-κB complex after the addition of cytokines. Furthermore, si*MANF* knockdown exacerbated *BCL10* expression in cytokine-treated EndoC-βH1 cells. These observations indicate that MANF interferes with the NF-κB pathway activation in beta cells upon cytokine treatment.

Interestingly, RNA-seq data showed a global increase in gene expression when the islets were stimulated with cytokines together with MANF. However, addition of MANF alone did not have an effect on the amount of mRNA measured. One of the potential mechanisms could be a reduction of mRNA degradation via suppression of the IRE1-dependent decay of mRNA. Supporting this, prenatal IRE1 activation has been detected in the MANF knockout mice since *sXbp1* expression is already upregulated at E18.5 [11]. Further studies will be necessary to elucidate the relationship between MANF and IRE1-dependent decay of mRNA regulation.

Most growth factors that stimulate beta cell proliferation in mice have not been effective in human cells. One important confounding factor is the age of the organ donors. It is well characterised that the proliferative capacity of beta cells declines with age due to the accumulation of the cell cycle inhibitor p16^Ink4a^ [27, 28]. It was recently shown that TGF-β inhibitors repress the Ink4a/Arf locus, resulting in increased beta cell proliferation in mice and transplanted human islets [29]. Interestingly, we saw an increase in beta cell proliferation when adding both TGF-β inhibitor and MANF, but not when either one alone was used. One explanation for this could be that the TGF-β inhibitor rejuvenates beta cells, making these cells more responsive to mitogens. Importantly, we were also able to verify the mitogenic effect of MANF in the human EndoC-βH3 model.

Several studies have shown that MANF expression and secretion are increased in situations of cellular stress [38, 39]. Furthermore, it has recently been shown that MANF levels are elevated in individuals newly diagnosed with type 1 diabetes [19]. However, based on studies in INS-1E cells, it was recently reported that inflammatory cytokines induce proteasomal degradation of MANF, making the beta cells more prone to apoptosis [12]. In our study, we found that cultured human beta cells persistently expressed and secreted up to threefold more MANF when exposed to cytokines associated with the pathogenesis of type 1 diabetes. In addition, MANF protein levels were increased despite a global inhibition of protein synthesis that normally occurs during ER stress, suggesting that MANF protein synthesis somehow bypasses PERK–eIF2α-induced translational inhibition. It is likely that secreted MANF exerts its protective effect in an autocrine/paracrine manner. Taken together, it appears that the amount of MANF is crucial for beta cell survival. Further supporting this, MANF expression is reduced in diabetes-susceptible mice compared with diabetes-resistant mice [40].

In conclusion, we have shown that recombinant MANF partially protects human pancreatic beta cells against proinflammatory-cytokine-induced cell death. Mechanistically, this is at least partially mediated through MANF inhibiting the NF-κB pathway and *BCL10*. Furthermore, MANF may enhance human beta cell proliferation. Our study elucidates the role of MANF as a protective and mitogenic growth factor for adult human beta cells that could potentially be used to prevent or reverse beta cell loss in diabetes.

## Electronic supplementary material

ESM (PDF 9.58 mb)

ESM Table 4 (XLSX 43 kb)

## Data Availability

All original data are available from the corresponding author on reasonable request.

## Abbreviations

EdU: 5-Ethynyl-2′-deoxyuridine
eIF2α: Eukaryotic initiation factor 2 alpha
ER: Endoplasmic reticulum
FC: Fold change
FDR: False discovery rate
GO: Gene Ontology (consortium)
IRE1: Inositol-requiring enzyme 1
MANF: Mesencephalic astrocyte-derived neurotrophic factor
PERK: Protein kinase RNA-like endoplasmic reticulum kinase
PFA: Paraformaldehyde
PDI: Protein disulfide isomerase
PI: Propidium iodide
qRT-PCR: Quantitative reverse transcription PCR
RNA-seq: RNA sequencing
si*MANF*: siRNA targeting *MANF*
siNT: Non-target siRNA
siRNA: Small interfering RNA
UPR: Unfolded protein response
