# Supplementary material for: MANF protects human pancreatic beta cells against stress-induced cell death
Source: Diabetologia. 2018 Jul 21;61(10):2202–14. doi: 10.1007/s00125-018-4687-y (PMC6133171; doi:10.1007/s00125-018-4687-y)
Supplement: Supplementary file 1 — (PDF 9.58 mb) [file 125_2018_4687_MOESM1_ESM.pdf]

Electronic Supplementary Material

ESM Methods

EndoC-βH1 cells

EndoC-βH1 cells [1] were cultured in low-glucose (1g/L) Dulbecco's modified Eagle's medium (DMEM; Sigma-Aldrich, Darmstadt, Germany) containing L-glutamine and sodium pyruvate, supplemented with 2% BSA fraction V (Roche Diagnostics, Mannheim, Germany), 50 µM 2-mercaptoethanol (Gibco, Invitrogen), 10 mM nicotinamide (Calbiochem, San Diego, CA, USA), 5.5 µg/ml transferrin (Sigma-Aldrich), 6.7 ng/ml selenite (Sigma-Aldrich), 100 U/ml penicillin and 172 µmol/l streptomycin. Cells were seeded on Matrigel (1%) / fibronectin (2 µg/ml) (Sigma-Aldrich) coated plates and cultured at 37°C and 5% CO_2.

EndoC-βH3 cells

EndoC-βH3 cells cell were obtained from Univercell Biosolution S.A.S. France and cultured as per their instructions [2]. Cells were maintained in OPTIβ1 complete medium containing 10 µg/ml puromycin (Ant-pr-1, Invivogen). Efficient excision of immortalizing transgene was achieved as per manufacturer's instruction with three weeks treatment of 1µM 4-Hydroxy Tamoxifen (4-OHT) (Sigma-Aldrich) and confirm by immunocytochemistry of SV40 (Calbiochem) and insulin. The proliferation experiments were performed in OPTIβ1 complete medium containing puromycin and 4-OHT as described above for 72h. Cells were fixed in 4% PFA for 20 min and EdU detected by Click-it Edu Alexa Fluor 488 Imaging kit (Invitrogen) according to manufacturer's protocol followed by SV40 staining. The proliferative EndoC-βH3 was counted, which is EdU positive and SV40 negative from 3-5 chosen

focal points of two experiments followed by counting total nucleus. Each image contained approximately 200-1000 cells.

Immunostaining and Immunoblotting of EndoC- β H1 cells

Immunohistochemistry was carried out as previously described [3]. In brief, the human pancreatic sections were deparaffinized and rehydrated using routine protocols. Sections were then treated with 1 mM EDTA buffer (pH 8) in a microwave oven to reveal the antigenic sites. Blocking was done using Ultra V Block (Thermo Scientific, Waltham MA, USA) for 10 min at RT to block non-specific binding sites followed by overnight incubation at 4°C with primary antibodies (ESM Table 2) diluted in PBS containing 0.1% Tween 20 (vol./vol). Nuclear staining was performed with DAPI (Vector Laboratories, Burlingame, CA, USA). Triple staining without nuclear staining were mounted with Vectashield (Vector Laboratories).

EndoC-βH1 cells were cultured on Matrigel and fibronectin coated 24-well *TPP* tissue culture plate or on glass coverslips and immunostained as described previously [4]. For primary antibodies see ESM Table 2. Hoechst 33342 was used to counterstain the nuclei. Images were acquired under EVOS fluorescence microscope (Life Technologies) or Zeiss AxioImager-3 microscope with Apotome and processed using Adobe Photoshop and ImageJ software.

Equal proteins (25 µg) were resolved by Any kD Mini-Protean -TGX gel (Bio-Rad), immunoblotted with corresponding antibodies as described in ESM Table 2 as described previously [4]. Membranes were incubated with species-specific HRP-linked secondary antibodies (1:5000) and visualization was performed following ECL

exposure with ChemiDoc XRS+ system and Image Lab Software (BioRad). A loading control was performed on the same blot for all western blot data. For MANF WB, cells were directly incubated with Laemmli buffer on ice for 30 min followed by 5 min heating at 95 °C then separated by SDS-PAGE. Densitometric analysis of bands from image were calculated using Image J (Media Cybernetics) software and intensities compared as phospho-p65 to actin; phospho-ERK and phospho-AKT to tubulin and MANF to GAPDH.

Quantitative RT-PCR

cDNA was synthesized using the random hexamer priming of the High Capacity cDNA Reverse Transcription kit according to the manufacturers recommendations (Applied Biosystems, Foster City, CA). The method for quantitative RT-PCR has been described previously [5]. Briefly, SYBR Green JumpStart Taq Ready Mix for quantitative PCR (Sigma-Aldrich) was used for the reactions with a Corbett Rotor-Gene 6000 (Qiagen, Hilden, Germany). The reactions were pipetted with a liquid handling system (Corbett CAS-1200, Qiagen). All reactions were performed in duplicates on at least three biological replicates. The median C_t values were used for $2^{-\Delta\Delta Ct}$ analysis. Cyclophilin G was used as an endogenous control. An exogenous positive control was used as a calibrator between all the real-time PCRs. Primer sequences for *MANF, GRP78* (also known as *HSPA5*), *CHOP* (also known as *DDIT3*), *sXBP1, ATF4, ATF6, ATF3, PreINS, INS, PDX1, MAFA,* CyclophilinG (also known as *PPIG*), *BCL10, Ki67, CDK1* and *CDK4* are presented in ESM Table 3.

MANF Assay

For the quantitation of secreted MANF protein, the conditioned medium samples from human islets and EndoC-βH1 cells were centrifuged at 5000 rpm for 5 min, and the supernatants were analyzed on an in-house sandwich ELISA specific for human MANF [6]. The dynamic range of the assay was 62.5 to 2000 pg/ml and the sensitivity was 45 pg/ml. Intra- and inter-assay coefficients of variation were 8.1% and 5.5%, respectively. For the measurement of cellular MANF cells were lysed with non-SDS lysis buffer TETG solution contains 20 mM Tris pH 8.0; 0.1% Triton X-100; 1% Glycerol; 137 mM NaCl; 2 mM EGTA and anti-protease tablet (Roche) for 30 min on ice. The lysate was next centrifuge at 5000 rpm for 5 min and store at -20 °C until MANF ELISA and BCA assay for total protein measurement. Total cellular MANF is presented as ng /ml /1.5 x 10^5 seeded cells after value corrected for the total protein content.

Quantification of Annexin-V / Propidium Iodide staining in EndoC-βH1 cells

Cells were harvested with accutase and pelleted by centrifugation (500g, 4 min.). Apoptotic cells were quantified with BD Annexin-V: FITC Apoptosis Detection Kit (#556547, BD Biosciences, San Jose, CA, USA) following manufacturer's instructions. Cells were analyzed by flow cytometry, using BD Accuri C6 (BD Biosciences) and at least 10,000 events were collected per sample. Data was analyzed using BD Accuri C6 analysis software (version 1).

RNA Sequencing using STRT protocol

For RNA sample collection, the islets were cultured in suspension in non-adherent plates in Ham's F10 medium supplemented with 0.5% BSA, penicillin (100 IU/ml) and streptomycin (100 µg/ml). Total RNA from human islets was isolated using the

RNeasy mini kit (Qiagen) according to the manufacturer's instructions. The samples used for transcriptome analysis were evaluated by Bioanalyzer 2100 (Agilent, Santa Clara, CA, USA). The RNA integrity number (RIN) values for all samples was >8.0. High-quality total-RNA (10 ng) was taken from the islets of six organ donors from the conditions described above (control, MANF, four cytokines and four cytokines with MANF) and processed according to highly multiplexed Single-cell Tagged Reverse Transcription (STRT) RNA sequencing method [7, 8]. Instead of processing as single cells, the method was used for generating barcoded cDNA libraries from low quantity (10ng) RNA samples. Poly-dT primers were used to enrich polyA+ mRNAs for sequencing from total RNA samples. High-quality 10 ng total-RNA was extracted from the islets of six organ donors from four conditions: control, MANF, four cytokines and four cytokines with MANF. The samples were processed with Single-cell Tagged Reverse Transcription (STRT) RNA sequencing protocol [7, 8] with minor modifications. Briefly, one microliter of total-RNA (10 ng/µl) samples were added to forty-eight barcoded plate where four microliters of cell capture buffer, contained 0.1% Triton X-100, 800 nM T30-VN- oligo, 2 mM deoxy-nucleotide (dNTP) mix, and 2 mM template-switching oligonucleotide (TSO) without magnesium chloride, was previously prepared. ERCC spike-in Mix A was diluted 1,000 times before adding 1 µl to 48-plex reverse transcriptase master mix. After cDNA synthesis, all 48 cDNAs were pooled into one 2-mL tube using 10% PEG-6000 and 0.9 mol NaCl (final concentration). The purified cDNA was first amplified using 14 cycles of PCR and later an additional 10 cycles to introduce a complete set of adapters for Illumina single read sequencing. Ready library was size- selected using sequential AMPure XP bead selection protocol where 0.73 and 0.223 bead/PCR

product ratios were used. Ready library was analyzed on three lanes of Illumina HiSeq2000 instrument.

RNA sequencing data processing and gene expression analysis

Data processing of the sequenced RNA libraries was performed using the STRTprep version v3dev pipeline (https://github.com/shka/STRTprep; commit 6389622). Briefly, the reads were de-multiplexed into individual samples using the sample-specific barcodes. Redundant reads were excluded according to unique molecular identifiers (UMIs), then mapped to the human genome assembly hg19/GRCh37 with RefSeq annotations [9] using Bowtie v. 1.1.0 [10] and Tophat v.2.0.12 [11]. For quality control, samples with low mapped read counts (< 200,000 reads/sample), high redundancy (>10), shallow spike-in counts (< 700 reads/sample), low spike-in map rate (< 90%), and low map rate to transcript start sites (< 70%) were excluded from subsequent analyses. The read counts were normalized to relative amounts compared to total spike-in counts. Differential expression analysis was performed using SAMstrt [12]. In addition to differential expression significance between control and patient samples, only transcripts with more biological variation than the background technical noise was considered as significant. Variation caused by technical noise was estimated from technical replicates using a generalized linear model with a gamma distribution, as described in [8].

For identification of genes and pathways specific to MANF rescue effects, a different pipeline was used. Mapped RNA reads were obtained from the pipeline as described in the previous section. Genes with low counts in more than three samples were removed before the identification of differentially expressed genes using the Bioconductor package edgeR v3.14.0 [13]. A false discovery rate (FDR) method was

employed to correct for multiple testing. Differential expression was defined as |log2 (ratio) | ≥ 2 (±4-fold) with the FDR set to 0.05. For pathway analysis, the Ingenuity Pathways Knowledge Base (Ingenuity Systems, CA, USA) was used.

References

1. Ravassard P, Hazhouz Y, Pechberty S, et al (2011) A genetically engineered human pancreatic β cell line exhibiting glucose-inducible insulin secretion. J Clin Invest 121:3589–3597

2. Benazra M, Lecomte M-J, Colace C et al (2015) A human beta cell line with drug inducible excision of immortalizing transgenes. Mol Metab 4:916–925

3. Hakonen E, Ustinov J, Mathijs I et al (2011) Epidermal growth factor (EGF)-receptor signalling is needed for murine beta cell mass expansion in response to high-fat diet and pregnancy but not after pancreatic duct ligation. Diabetologia 54:1735–1743

4. Chandra V, Albagli-Curiel O, Hastoy B et al (2014) RFX6 regulates insulin secretion by modulating Ca2+ homeostasis in human β cells. Cell Rep 9:2206–2218

5. Hakonen E, Ustinov J, Eizirik DL et al (2014) In vivo activation of the PI3K-Akt pathway in mouse beta cells by the EGFR mutation L858R protects against diabetes. Diabetologia 57(5):970-9

6. Galli E, Härkönen T, Sainio MT et al (2016) Increased circulating concentrations of mesencephalic astrocyte-derived neurotrophic factor in children with type 1 diabetes. Sci Rep 6:29058

7. Islam S, Zeisel A, Joost S et al (2014) Quantitative single-cell RNA-seq with unique molecular identifiers. Nat Methods 11:163–166

8. Krjutškov K, Katayama S, Saare M et al (2016) Single-cell transcriptome analysis of endometrial tissue. Hum Reprod 31:844–853

9. Pruitt KD, Tatusova T, Brown GR, Maglott DR (2012) NCBI Reference Sequences (RefSeq): current status, new features and genome annotation policy. Nucleic Acids Res 40:D130–5

10. Langmead B, Trapnell C, Pop M, Salzberg SL (2009) Ultrafast and memory-efficient alignment of short DNA sequences to the human genome. Genome Biol 10:R25

11. Kim D, Pertea G, Trapnell C et al (2013) TopHat2: accurate alignment of transcriptomes in the presence of insertions, deletions and gene fusions. Genome Biol 14:R36

12. Katayama S, Töhönen V, Linnarsson S, Kere J (2013) SAMstrt: statistical test for differential expression in single-cell transcriptome with spike-in normalization. Bioinformatics 29:2943–2945

13. McCarthy DJ, Chen Y, Smyth GK (2012) Differential expression analysis of multifactor RNA-Seq experiments with respect to biological variation. Nucleic Acids Res 40:4288–4297

ESM Table 1 Donors

Donor	ID	Gender	Age (years)	BMI (kg/ m^2)	HbA1c % (mmol/mol)	Purity (%)	Cold ischemia time (h:min)	Research use
1	FFPE 1	female	25	-	-	-	-	Immunohistochemistry
2	FFPE 2	male	16	-	-	-	-	Immunohistochemistry
3	FFPE 3	male	65	-	-	-	-	Immunohistochemistry
4	D150213	male	61	26.3	5.3 (34)	39	10:44	Cytokines
5	H1880	male	72	24.7	5.3 (34)	63	12:53	Cytokines, Proliferation
6	H1882	female	72	33.3	5.7 (39)	87	10:53	Cytokines RNA seq, Proliferation
7	H1883	female	69	25.4	-	35	5:36	Proliferation
8	H1921	female	55	23.8	5.4 (36)	95	9:11	Cytokines RNA seq, Proliferation
9	H1922	male	56	25.8	5.5 (37)	73	-	Cytokines RNA seq, Proliferation
10	H1929	female	66	26.7	5.6 (38)	95	14:28	Cytokines
11	H1933	male	57	28.1	5.5 (37)	41	08:01	Cytokines
12	H1950	male	58	27.2	-	59	10:45	Cytokines RNA seq
13	H1955	male	78	-	5 (31)	93	-	Cytokines RNA seq
14	H1956	unknown	67	28.1	-	53	5:16	Cytokines
15	H1966	female	54	24.2	5.3 (34)	68	5:32	Cytokines, Proliferation
16	H1969	male	76	25.5	-	85	07:12	Cytokines RNA seq, Proliferation
17	H1971	male	69	18	4.8 (29)	77	07:50	Proliferation
18	H1974	male	66	23.9	5.5 (37)	72	09:41	Proliferation
19	H1982	male	60	29.7	5.8 (40)	67	06:20	Proliferation

ESM Table 2 Antibodies

Peptide/protein target	Manufacturer; catalog #	Species raised in	Dilution
MANF	Icosagen; 310-100	Rabbit; polyclonal	1:300
Insulin	Dako Cytomation; A0564	Guinea pig; polyclonal	1:1000
Glucagon	Sigma; G2654	Mouse; monoclonal	1:500
PP	Sigma; SAB2500747	Goat; polyclonal	1:500
PDI	Enzo/AH Diagnostics; ADI-SPS-891-F	Mouse; monoclonal	1:200
GM130	BD Transduction Laboratories; 610823	Mouse; monoclonal	1:200
C-peptide	Cell Signaling Technology; #4593	Rabbit; polyclonal	1:100
Cleaved Caspase-3	Cell Signalling; #9661	Rabbit; polyclonal	1:250
RELA	Santa-Cruz; #sc8008	Mouse; monoclonal	1:200
phospho p65 (Ser536)	Cell Signaling; #3033	Rabbit; monoclonal	1:1000
Tubulin	Sigma; T5168	Mouse; monoclonal	1:2000
MANF (WB)	Icosagen; 310-100	Rabbit; polyclonal	1:500
GAPDH	MAB374, Millpore	Mouse; monoclonal	1:300
Phospho ERK 1/2	Cell Signaling; #4370	Rabbit; monoclonal	1:1000
ERK 1/2	Cell Signaling; #4695	Rabbit; monoclonal	1:1000
Phospho AKT(T308)	Cell Signaling; #9275S	Rabbit; polyclonal	1:1000
β -actin	Sigma; A5441	Mouse; monoclonal	1:5000
SV40	Millipore; #PAb416	Mouse; monoclonal	1:100

ESM Table 3 Primers

Target cDNA	Origin	Sequence of 5'-primer (F)	Sequence of 3'-primer (R)
MANF	hum (NM_006010.4)	GGCGACTGCGAAGTTTGTAT	TTGCTTCCCGGCAGAACTTT
GRP78 (*HSPA5*)	hum (NM_005347.4)	TGGCTGGAAAGCCACCAAGATGCT	GGGGGAGGGCCTGCACTTCCAT
CHOP (*DDIT3*)	hum (NM_001195053.1)	GCACCTCCCAGAGCCCTCACTC	CCCGGGCTGGGGAATGACCA
sXBP1	hum (NM_001079539.1)	CTGCTGAGTCCGCAGCAGGTGCA	GGTCCAAGTTGTCCAGAATGC
ATF4	hum (NM_001675.2)	AAGGCGGGCTCCTCCGAATGG	CAATCTGTCCCGGAGAAGGCATCC
ATF6	hum (NM_001675.2)	ACCTGCTGTTACCAGCTACCACCCA	GCATCATCACTTCGTAGTCCTGCCC
ATF3	hum (NM_001674.3)	AGAAAGAGTCGGAGAAGC	TGAAGGTTGAGCATGTATATC
PreINS	Ref #15	GTGAACCAACACCTGTGCGG	AGGGGCAGCAATGGGCAGTT
INS	hum (NM_000207)	TGTCCTTCTGCCATGGCCCT	TTCACAAAGGCTGCGGCTGG
PDX1	hum (U30329)	AAGTCTACCAAAGCTCACGCG	GTGCGCGTCCGCTTGTTCT
MAFA	hum (NM_201589)	GCCAGGTGGAGCAGCTGAA	CTTCTCGTATTTCTCCTTGTAC
CyclophilinG (*PPIG*)	hum (NM_004792)	TCTTGTCAATGGCCAACAGAG	GCCCATCTAAATGAGGAGTTG
**#***BCL10*	NM_003921	TGAAGAAGGACGCCTTAG	TTTTCCAGCCCTTTTTCTAC
Ki67	Reference #17		
CDK1	Reference #17		
CDK4	Reference #17		

#*Sigma KiCqStart Syber green primer*

ESM Fig.1

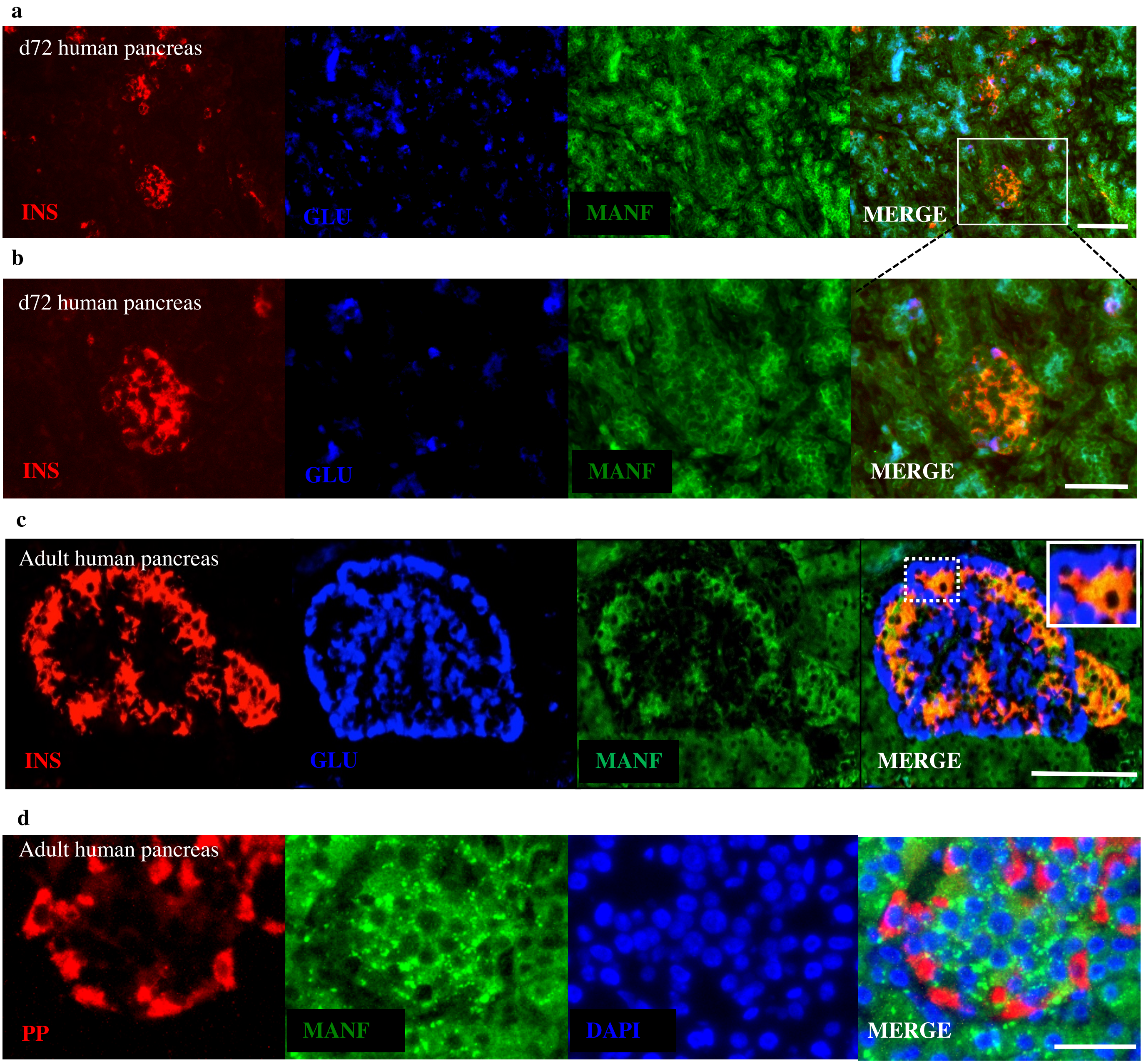

MANF immunoreactivity in human pancreas. (a-b) Immunohistochemical analysis of formalin fixed human fetal d72 pancreatic section stained for MANF (green) and counterstained with insulin (INS, red) and glucagon (GLU, blue). **(a)** MANF immunoreactivity can be detected throughout the pancreatic epithelium. Scale bar: 100 µm. **(b)** High magnification from (a) demonstrates co-localisation of MANF and INS. At this stage glucagon positive cells are also positive for INS and MANF. Scale bar: 50 µm. **(c)** Immunohistochemical analysis of formalin fixed human adult pancreatic section stained for MANF (green) and counterstained with insulin (INS, red) and glucagon (GLU, blue) showing that MANF co-localizes with insulin but not with glucagon in the adult pancreas. Scale bar: 100 µm. **(d)** Human adult pancreatic section stained for MANF (green) and counterstained with pancreatic polypeptide (PP, red) and DAPI (blue) showing that MANF does not co-localize with PP. Scale bar: 25 µm.

ESM Fig. 2

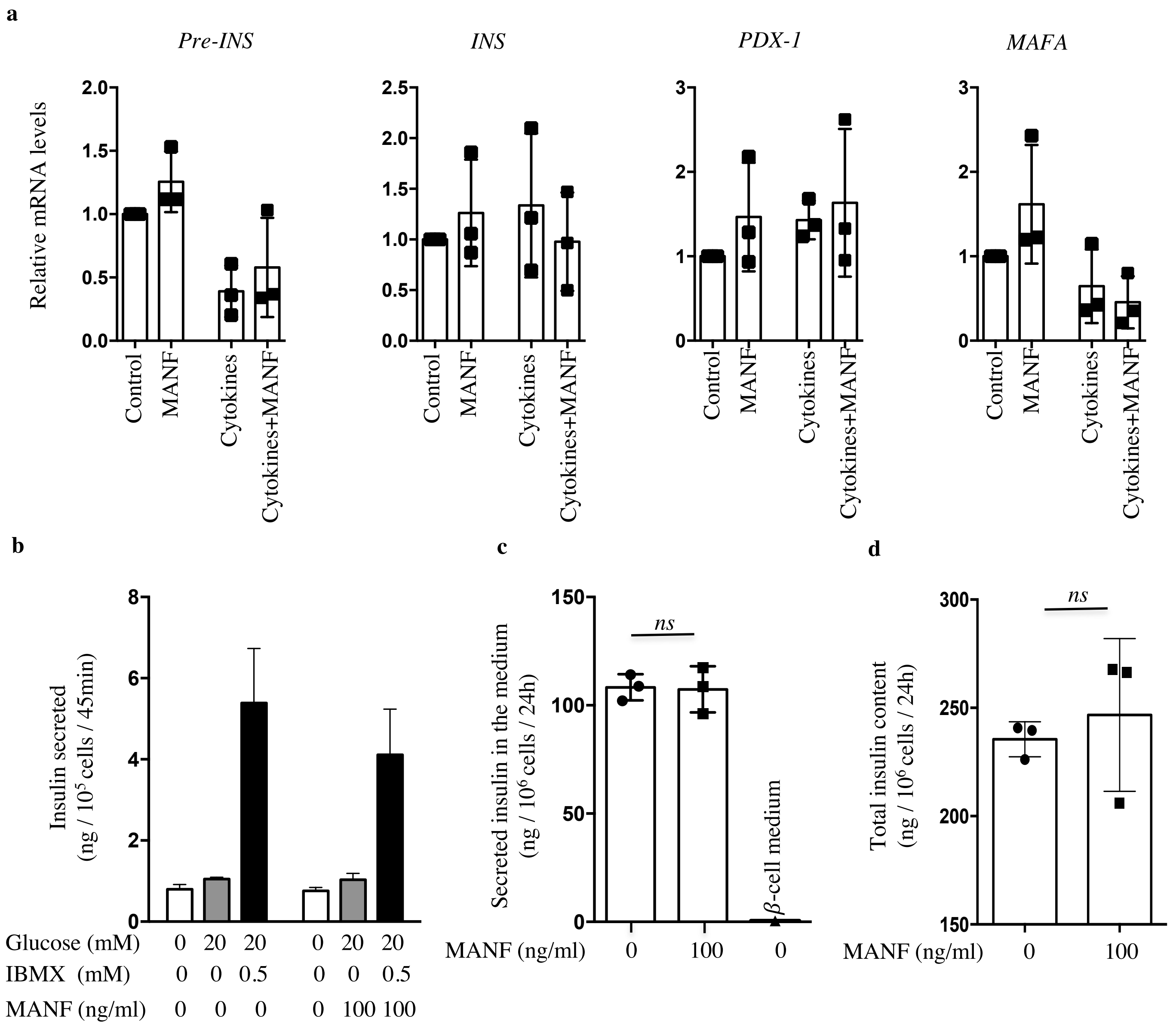

Effect of MANF on the expression of pancreas specific genes, insulin secretion and total insulin content. **(a)** qRT-PCR based analysis for the expression of beta cell specific genes *PreINS, INS, PDX1 and MAFA*. qRT-PCR data were normalized to housekeeping gene *Cyclophilin G* **(b)** Insulin secretion in response to 45 min incubations with 0mM glucose, 20mM glucose and 20mM glucose + 0.5mM IBMX in EndoC-βH1 cells in the presence or absence of MANF (100ng/ml). Data expressed as ng of secreted insulin per 45min per 10^5 cells (cells counted simultaneously in duplicate wells). **(c)** EndoC-βH1 cells were treated with MANF (100 ng/ml) for 24h and the secreted insulin in the supernatant was quantified by Elisa, **(d)** the total cellular insulin content was also quantified in similar experiment. Data represents mean values of at least 3 independent experiments and are presented as scatter plot of mean ± SD.

ESM Fig. 3

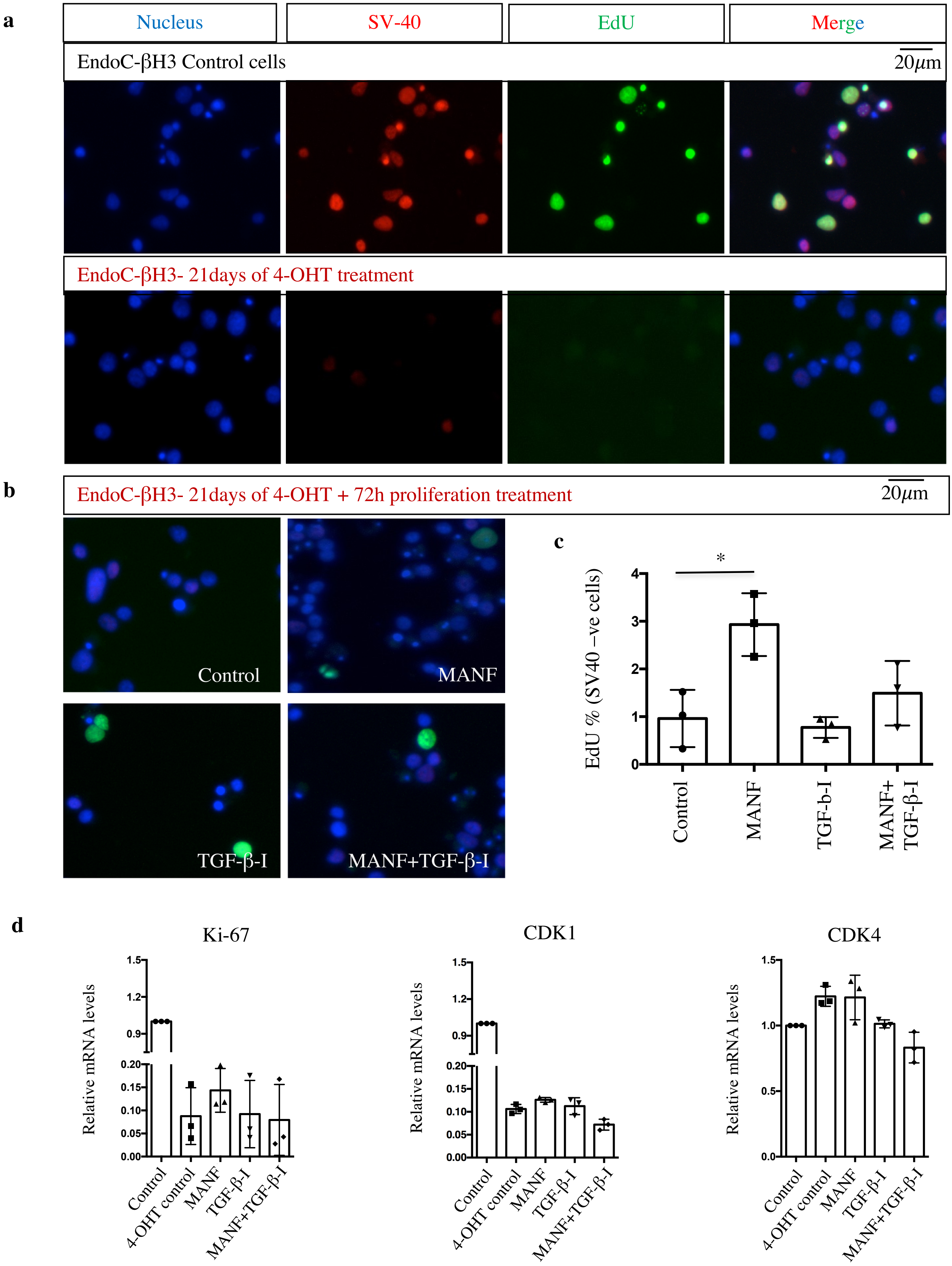

a
Nucleus
SV-40
EdU
Merge
EndoC-βH3 Control cells
20μm
EndoC-βH3- 21days of 4-OHT treatment
b
EndoC-βH3- 21days of 4-OHT + 72h proliferation treatment
20μm
Control
MANF
TGF-β-I
MANF+TGF-β-I
c
*
EdU % (SV40 –ve cells)
0
1
2
3
4
Control
MANF
TGF-b-I
MANF+ TGF-β-I
d
Ki-67
CDK1
CDK4
Relative mRNA levels
0.00
0.05
0.10
0.15
0.20
0.9
1.2
1.5
0.0
0.5
1.0
Control
4-OHT control
MANF
TGF-β-I
MANF+TGF-β-I

ESM Fig. 3

MANF induces proliferation of human β-cells. EndoC-βH3 cells were treated with 1 μM 4-hydroxy tamoxifen (4-OHT) for 21 days. **(a)** Control EndoC-βH3 (upper panel) and 4-OHT treated cells (lower panel) were stained for SV40LT (red) and EdU (green). Proliferation assays were performed at day 21. Such cells were cultured for 72 h with MANF (100 ng/ml), TGFβ inhibitor SB431542 (2 μM) or both. EdU was added to the culture medium at the onset of the experiment. **(b)** Representative image of EdU (green) and SV40LT (red) double immunofluorescence staining. **(c)** Quantification of proliferating β-cells analyzed by EdU expression. The cells stain negatively for SV40LT (red). Scale bar: 20 μm. (n=3) **(d)**. qRT-PCR based analysis for the expression of proliferation and cell-cycle related genes *Ki67, CDK1 and CDK4* in such experiment. qRT-PCR data were normalized to the housekeeping gene *Cyclophilin G*. Data are presented as mean ± SD of 3 technical replicates. *$p<0.05$; (one-way ANOVA, followed by Tukey's test).

ESM Fig. 4

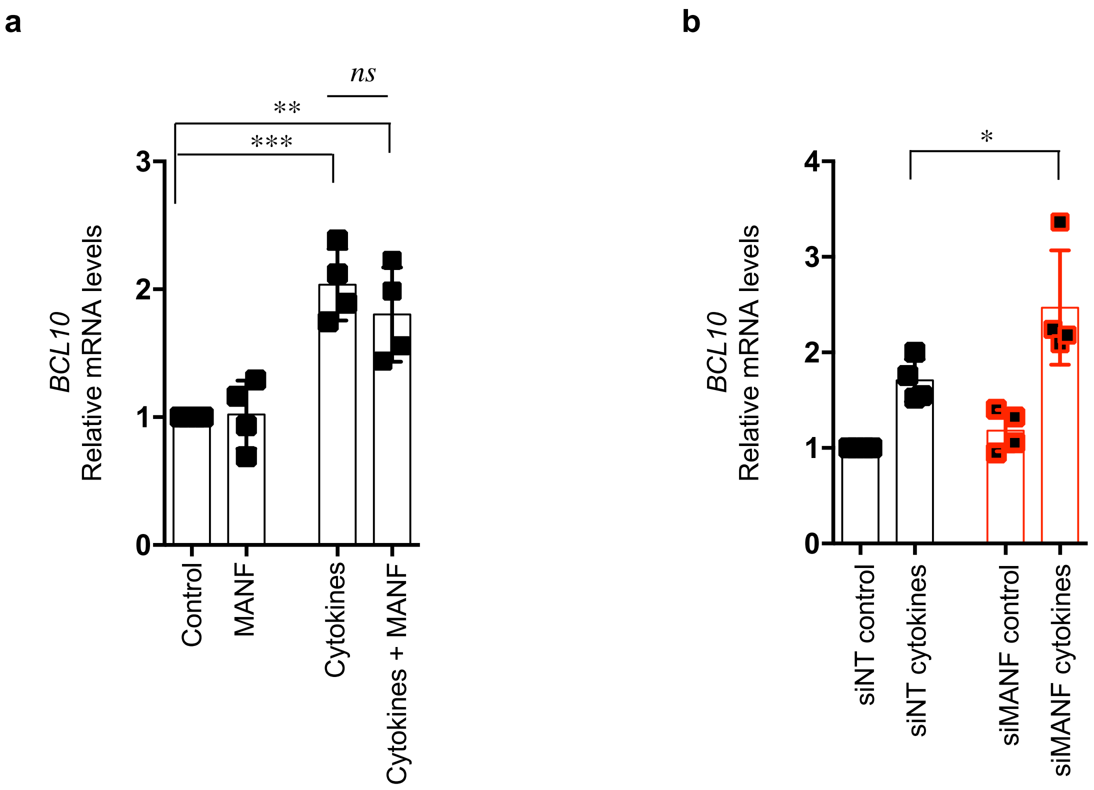

Effect of MANF on *BCL10* expression. **(a)** EndoC-βH1 cells were treated with MANF alone, cytokine cocktail II or cytokines II with MANF for 24 h. Expression of *BCL10* was determined by qRT-PCR. **(b)** EndoC-βH1 cells were transfected with siNT or siMANF for 72h, further exposed to cytokine cocktail II or left untreated for 24h and *BCL10* expression was determined. qRT-PCR data were normalized to C*yclophilin G* and plotted as fold over siNT- control cells. Data are presented as mean ± SD of 4 independent experiments. *p<0.05; **p<0.01 and ***p<0.001 (one-way ANOVA, followed by Tukey's test).

ESM Fig. 5

a

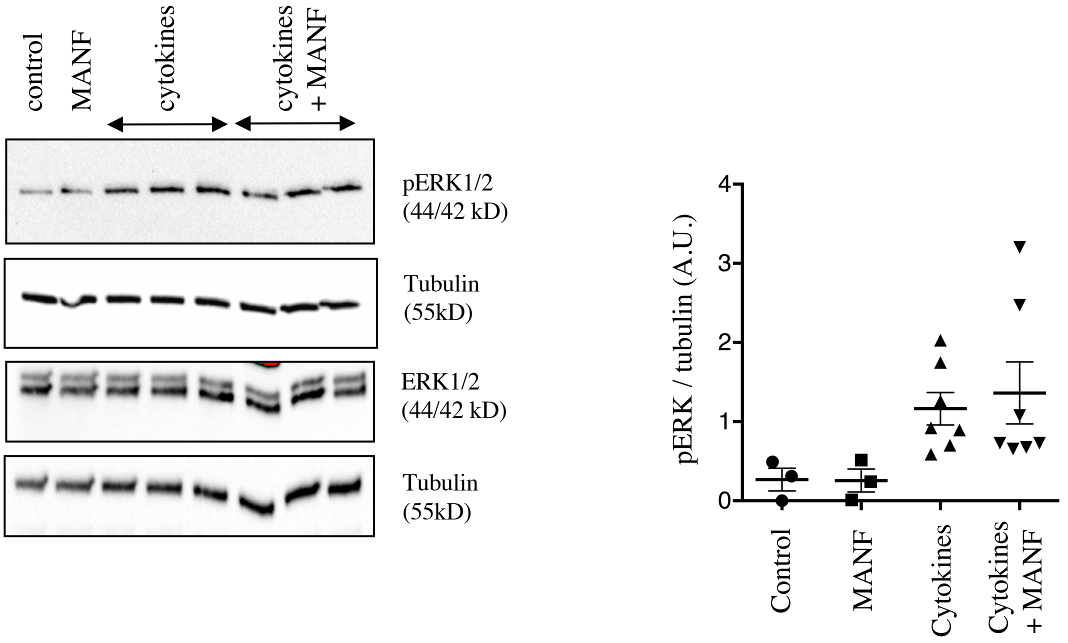

b

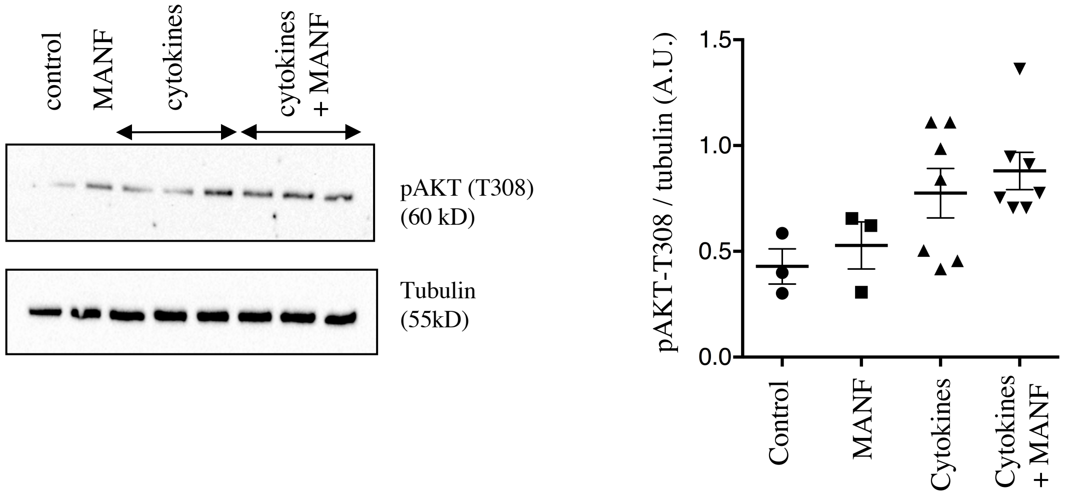

Effect of MANF on phosphorylation of ERK and AKT. EndoC-βH1 cells treated with MANF, cytokine cocktail II **or** cytokines cocktail II + MANF for 8h. **(a)** Western blot analysis of Phospho-ERK and total ERK with respective loading control tubulin **(b)** Phospho-AKT with loading control tubulin and their respective densitometric quantification of the bands normalized to tubulin. Data represent mean±SD of at least 3 independent experiments.
